# The burden of illness of osteoporosis in Canada

**DOI:** 10.1002/jbmr.1615

**Published:** 2012-08

**Authors:** J.-E. Tarride, R. B. Hopkins, W. D. Leslie, S. Morin, J. D. Adachi, A. Papaioannou, L. Bessette, J. P. Brown, R. Goeree

**Affiliations:** Programs for Assessment of Technology in Health (PATH) Research Institute, St Joseph’s Healthcare Hamilton, 25 Main Street West, Suite 2000, Hamilton, ON L8P 1H1, Canada. Department of Clinical Epidemiology and Biostatistics, McMaster University, Hamilton, ON, Canada; Programs for Assessment of Technology in Health (PATH) Research Institute, St Joseph’s Healthcare Hamilton, 25 Main Street West, Suite 2000, Hamilton, ON L8P 1H1, Canada. Department of Clinical Epidemiology and Biostatistics, McMaster University, Hamilton, ON, Canada; Department of Medicine, University of Manitoba, Winnipeg, MB, Canada; Department of Medicine, McGill University, Montréal, QC, Canada; Department of Medicine, McMaster University, Hamilton, ON, Canada; Department of Medicine, McMaster University, Hamilton, ON, Canada; Department of Medicine, Laval University, Quebec City, QC, Canada; Department of Medicine, Laval University, Quebec City, QC, Canada; Programs for Assessment of Technology in Health (PATH) Research Institute, St Joseph’s Healthcare Hamilton, 25 Main Street West, Suite 2000, Hamilton, ON L8P 1H1, Canada. Department of Clinical Epidemiology and Biostatistics, McMaster University, Hamilton, ON, Canada

**Keywords:** Burden of illness, Canada, Fragility fracture, Health economics, Osteoporosis

## Abstract

**Summary:**

To update the 1993 burden of illness of osteoporosis in Canada, administrative and community data were used to calculate the 2010 costs of osteoporosis at $2.3 billion in Canada or 1.3% of Canada’s healthcare expenditures. Prevention of fractures in high-risk individuals is key to decrease the financial burden of osteoporosis.

**Introduction:**

Since the 1996 publication of the burden of osteoporosis in 1993 in Canada, the population has aged and the management of osteoporosis has changed. The study purpose was to estimate the current burden of illness due to osteoporosis in Canadians aged 50 and over.

**Methods:**

Analyses were conducted using five national administrative databases from the Canadian Institute for Health Information for the fiscal-year ending March 31 2008 (FY 2007/2008). Gaps in national data were supplemented by provincial and community data extrapolated to national levels. Osteoporosis-related fractures were identified using a combination of most responsible diagnosis at discharge and intervention codes. Fractures associated with severe trauma codes were excluded. Costs, expressed in 2010 dollars, were calculated for osteoporosis-related hospitalizations, emergency care, same day surgeries, rehabilitation, continuing care, homecare, long-term care, prescription drugs, physician visits, and productivity losses. Sensitivity analyses were conducted to measure the impact on the results of key assumptions.

**Results:**

Osteoporosis-related fractures were responsible for 57,413 acute care admissions and 832,594 hospitalized days in FY 2007/2008. Acute care costs were estimated at $1.2 billion. When outpatient care, prescription drugs, and indirect costs were added, the overall yearly cost of osteoporosis was over $2.3 billion for the base case analysis and as much as $3.9 billion if a proportion of Canadians were assumed to be living in long-term care facilities due to osteoporosis.

**Conclusions:**

Osteoporosis is a chronic disease that affects a large segment of the adult population and results in a substantial economic burden to the Canadian society.

## Introduction

Osteoporosis Canada recently updated the 2002 clinical practice guidelines for the diagnosis and management of osteoporosis in Canada [1, 2]. The new guidelines [1] emphasize the need to assess for fracture risk in order to prevent the excess morbidity, mortality, and economic burden associated with osteoporosis and associated fragility fractures. While the direct economic burden of osteoporosis in Canada was estimated at $1.3 billion dollars in 1993 ($1.8 billion in 2010 dollars) [3], no recent study has updated these results despite the fact that many changes have occurred in patient demographics and disease management. Indeed, the Canadian population aged 50 and over has increased from 7.3 million in 1993 to 11.0 million in 2008 [4], and new risk assessment tools and treatment options have been introduced. To have a better understanding of the current economic burden of osteoporosis in Canada, we estimated the 2010 burden of illness of osteoporosis among Canadians aged 50 and over.

## Methods

### Overview

This prevalence-based burden of illness study was conducted using national, provincial, and community data. National data estimates were used if available. Gaps in national data were filled with provincial data extrapolated to the national level based on population demographics (i.e., age and sex). Sensitivity analyses were conducted to assess the impact of key assumptions on the estimates. All costs are presented in 2010 Canadian dollars and both a payer and a societal perspective were taken. When necessary, costs were inflated to 2010 using the Consumer Price Index of Statistics Canada [5].

### Data sources

Five data sets from the Canadian Institute for Health Information (CIHI) were used to gather Canadian data on acute care (Discharge Abstract Database—DAD) [6], emergency visits (National Ambulatory Care Reporting System—NACRS) [7], same day surgery (NACRS for Ontario), rehabilitation services (National Rehabilitation Reporting System—NRS) [8], home care (Home Care Reporting System—HCRS) [9], and continuing care (Continuing Care Reporting System—CCRS) [10]. IMS Health [11] and Brogan Inc. [12] provided data to estimate osteoporosis-related physician and prescription drug costs. Patient and caregiver productivity losses were calculated using data from the Canadian Multicentre Osteoporosis Study (CaMos) [13] and Statistics Canada [14, 15]. In addition to these national data sources, fracture data from the Recognizing Osteoporosis and Its Consequences in Quebec (ROCQ) program [16], from the Resident Assessment Instrument for Home Care (RAI-HC) of Ontario, and from the Manitoba Centre for Health Policy (MHCP) [17] were used to fill gaps or to check results for consistency.

### Identification of fractures and attribution to osteoporosis

For the fiscal year April 1, 2007 to March 31, 2008 (FY 2007/2008), fractures in Canadians 50+ were identified in CIHI databases using two definitions: [1] most responsible diagnosis code at discharge of fracture (ICD-10 CA) (see Appendix 1 for a list of codes) or [2] a combination of a secondary code for fracture and an intervention indicative of treatment for a fracture (e.g., fixation, immobilization, reduction, partial excision, repair). The most responsible diagnosis for a patient’s stay in hospital is established at discharge and corresponds to the one diagnosis or condition that can be described as being the most responsible for the patient’s stay. Fracture records associated with a severe trauma code were excluded from the base case analyses. All low-trauma hip and vertebral fractures were attributed to osteoporosis (i.e., 100%). The rate of attribution to osteoporosis for wrist, humerus, other, and multiple fractures was derived from Mackey et al. [18] In Mackey et al., the percentages of low-trauma fractures occurring in individuals with low bone mineral density were 74.5% for men and 90.4% in women. A 56.0% attribution rate of osteoporosis for non-hip non-vertebral fractures (*X*) in men was obtained by solving the following equation with respect to *X*: (number of hip and vertebral fractures in men×100% osteoporosis attribution rate + number of non-hip non-vertebral fractures in men×*X*% osteoporosis attribution rate)/(total number of fractures in men)=74.5% as per Mackey et al.’s results for men. The same exercise was repeated in women to derive an 81.5% attribution rate of osteoporosis for non-hip non-vertebral fractures.

### Estimation of the costs associated with hospitalizations, emergency room visits, and same day surgeries

DAD covers all admissions to acute care hospitals in Canada with the exception of Quebec; Quebec data were therefore extrapolated. Given that Ontario is the only province for which all emergency care visits and same day surgeries are reported in NACRS, the data from Ontario were extrapolated to the national level based on population characteristics. The resource intensity weights (RIW) [19] recorded for each individual were used to assign costs to hospital-stay admissions, emergency room visits, and same day surgeries. RIWs, which are assigned to each patient on discharge, estimate the relative amount of resources needed for a specific admission. Although different RIWs apply to each fracture type, the value of the RIW depends on the Case Mix Group—a Canadian patient classification system assigning similar inpatient cases to a single group—to which they are assigned as well as other factors that affect resource utilization and length of stay (e.g., age, comorbidity levels). Since the RIW does not include the costs related to physician visits (e.g., orthopedic surgeons, anesthesiologists, radiologists), diagnostic tests (e.g., X-rays), and procedures (e.g., fixation), these costs were added to RIW costs to determine the total cost of an admission, emergency visit, or same day surgery (i.e., for each patient). The number of physician visits/assessments per admission was derived from the length of stay and costed in function of the fee structure given in Table 1. For example, the value of one physician visit at admission was $79.20 while a cost of $55.45 was applied to the visit during the second day of hospitalization (Table 1). Table 1 also presents the detailed unit costs associated with the RIW, diagnostics, and procedures.

### Estimation of the costs associated with rehabilitation, continuing care, long-term care, and home care

Since NRS and CCRS databases do not report the most responsible diagnosis, DAD was used to identify how many individuals were transferred from acute care to rehabilitation, continuing care, or long-term care facilities. Since the main reason for admission to these facilities prior to the admission was unknown (i.e., not osteoporosis-related), individuals already residing in rehabilitation, continuing care, or long-term care facilities prior to the acute care admission were excluded from the base case analyses in order to be conservative in our estimates. As such, only the excess number of individuals discharged to a particular destination (e.g., number of men discharged to long-term care facilities minus number of men originating from long-term care facilities) was used in the cost calculations. The CIHI–HCRS database on home care in Ontario was used to extrapolate how many Canadians received home care services for osteoporosis-related fractures. Manitoba data were used to estimate the length of stay in long-term care and time receiving home care services following a fracture. All the extrapolations to the national level were adjusted by age and sex.

The costs associated with rehabilitation and continuing care were calculated by multiplying the excess number of individuals transferred from acute care to rehabilitation or continuing care facilities, respectively, by the average NRS and CCRS’s RIW inflated for physician visits. Based on Ontario data, daily costs of $24 and $148 were applied to home care services and long-term care, respectively (Table 1).

### Estimation of physician and prescription drug costs

The number of physician visits due to osteoporosis was derived from the IMS Health Canada physician survey which is designed to provide information about disease and treatment patterns of physicians in Canada. This sample includes 652 physicians stratified by region and representing all major specialties. Each calendar quarter, the physician reports on all patient contacts for a period of two consecutive days. Physician visit fees were applied to the IMS data according to the Ontario Schedule of Benefits for Physician Services [20]. Costs associated with osteoporosis-related prescription drugs (e.g., alendronate, etidronate, risedronate, zoledronic acid, teriparatide, raloxifene, and calcitonin) were derived from Brogan Inc. Public and private drugs claims collected at pharmacies are adjudicated online and transmitted monthly to IMS Brogan under a data service agreement with the Canadian provincial governments and private drug plans. IMS Brogan covers 100% and 65% of all public and private drug claims in Canada, respectively. Private drug claims were extrapolated to national levels. IMS and Brogan data were provided by Amgen Canada.

### Estimation of indirect costs

To reflect a societal perspective, time lost from work following an osteoporosis-related fracture and caregiver wage loss were valued. To estimate the productivity losses, the number of days spent in acute and non-acute care (e.g., rehabilitation) was first estimated for individuals aged 50 to 69 using CIHI data. This number was multiplied by the labor force participation rate (i.e., 77% of individuals aged 50 to 59 and 45% of individuals aged 60 to 69 [15]) and by the Canadian average daily wage for that age group ($24.12 per hour×8 h per day) [14]. Based on CaMos [21] and CIHI data, the value of caregiver wage loss was calculated by multiplying the number of osteoporosis-related admissions by the percentage of patients using caregivers (47.2%) times the number of days of care (37 days) times the percentage of caregivers being employed (35.8%) times the average daily wage ($24.12 per hour×8 h).

### Sensitivity analyses

Several sensitivity analyses were conducted to assess the impact of key assumptions on the burden of illness estimates. First, the assumptions related to the attribution to osteoporosis in women were changed by using Quebec data on fragility fractures among 2,075 women 50 years and older (e.g., 75.7% between the ages of 50 to 59 years old to 91.8% in the group over the age of 80) [22]. Second, although we identified individuals who were hospitalized with a most responsible diagnosis code of osteoporosis but without a diagnosis of fracture or intervention code, the base case analysis excluded those individuals, as we were uncertain how to attribute the admission. In an additional sensitivity analysis, we included these cases in our cost estimates. Third, in the absence of accurate data on the reasons for admissions to long-term care facilities, the primary analysis ignored the costs associated with those individuals residing on a yearly basis in long-term care facilities due to osteoporosis. Based on an economic model developed for the Ontario Ministry of Health and Long Term Care’s Medical Advisory Secretariat [23], it was estimated that 17% of men and 21% of women over the age of 65 were residents in long-term care facilities following an osteoporosis-related fracture. Finally, the last sensitivity analysis was conducted assuming that all high and low-trauma fractures were due to osteoporosis. This scenario was based on the evidence generated by Mackey et al. showing that low BMD predicts both high and low-trauma fractures [18] and that antiresorptive treatments prevent high- and low-trauma fractures [24], leading to the recommendation for using all fractures as standard outcomes in osteoporosis trials and observational studies.

## Results

### Hospitalizations, same day surgeries, and emergency room visits due to osteoporosis-related fractures

**Fig. 1:**
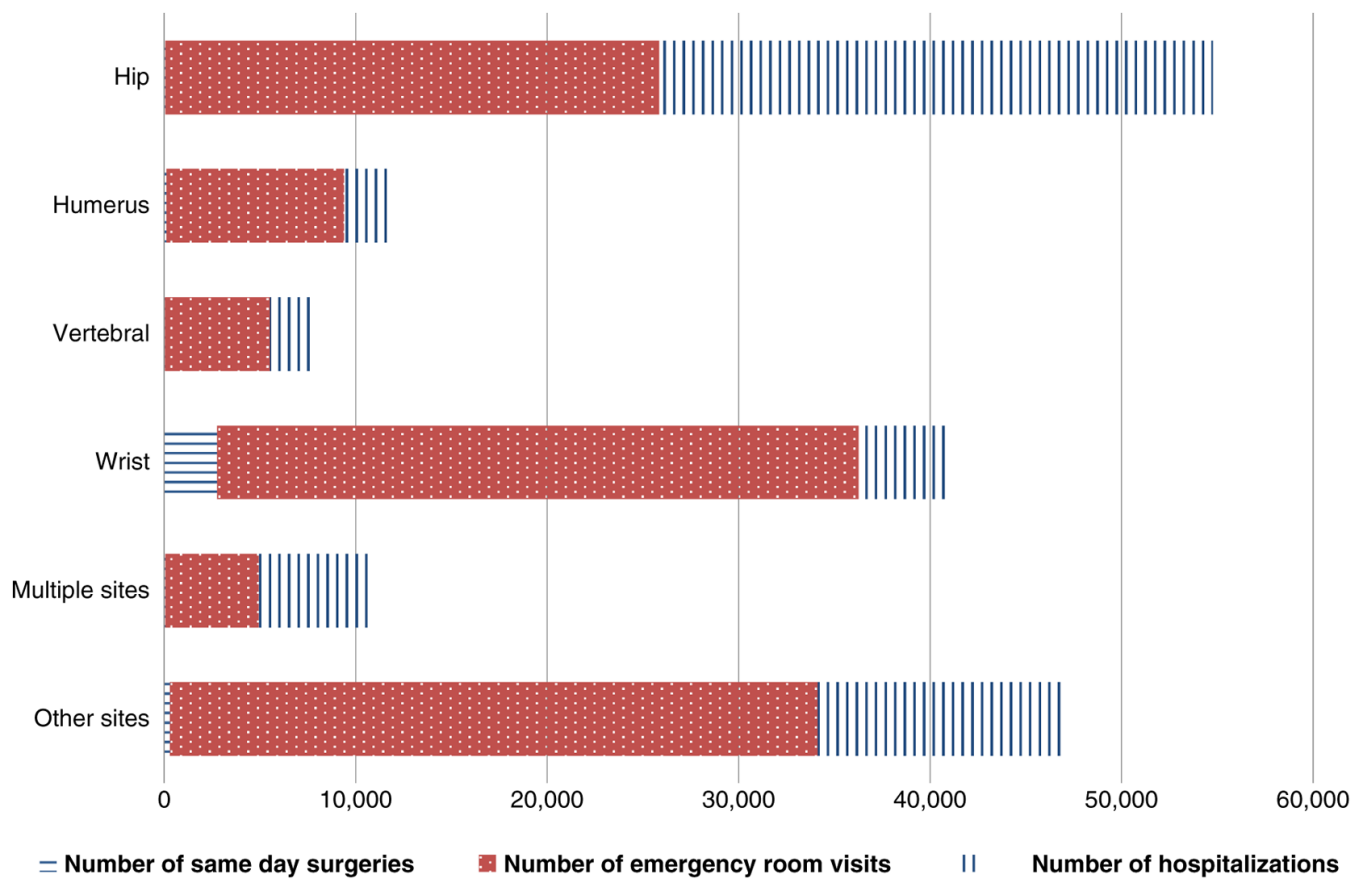
Number of same day surgeries, emergency room visits, and hospitalizations by type of osteoporosis-related fracture in Canada in fiscal year 2007/2008 (independent of discharge destination)

As shown in Table 2, CIHI data for all Canadian provinces except Quebec indicated that 44,707 hospitalizations were attributable to osteoporosis-related fractures in FY 2007/ 2008. The number of osteoporosis-related fractures in Quebec was estimated at 12,706 for a total of 57,413 hospitalizations in Canada. These hospitalizations resulted in 832,594 hospitalized days. The mean length of stay was 14.5 days [median (Q1, Q3)=7 (1, 0.15) days]. Fractures in women accounted for approximately 70% of all hospital admissions (men—16,855; women—40,550) and hospitalized days (men—228,231; women—604,363). Among women, hip fractures accounted for half of the hospitalized days (316,607 out of 604,363). Over 70% of all fractures occurred in individuals older than 70 years with the highest number of hospitalizations observed in the 81–90 years age group (21,033 of 57,413). In addition, osteoporosis-related fractures resulted in 112,740 emergency room visits and 3,433 same day surgeries. Eighty percent of all same day surgeries were due to wrist fractures while wrist (30%), hip (23%), and other fracture sites (30%) accounted for more than 80% of all osteoporosis-related fracture visits to the ER (Fig. 1).

### Costs associated with hospitalizations, same day surgeries, and emergency room visits

The costs associated with osteoporosis-related fracture hospitalizations, same day surgery, and emergency visits in FY 2007/2008 were estimated at $1.2 billion, of which 85% was due to hospitalizations (Table 3). Fractures in women and of the hip accounted for 71% and 53% of the acute care costs, respectively (Table 3). Hospitalizations following fractures at multiple sites and hip fractures were the most costly at approximately $23,404 and $20,163 per hospitalization, respectively. The average cost of a hospitalization for wrist fracture was the lowest at $8,848 while humerus, vertebral, or other sites-related hospitalizations cost approximately $13,000. Hospitalization costs increased with age (e.g., from $11,434 in women aged 50–59 to $19,456 in women aged 80–89). The increased costs were driven by longer lengths of stay (e.g., an average of 7.8 days for individuals aged 50–59 versus 17.4 days for 80–89 years old). No major differences between fracture sites or age groups were observed in terms of costs associated with same day surgery ($3,166 to $4,238) or emergency room visits (e.g., $816 to $1,913).

### Costs associated with rehabilitation, continuing care, long-term care, and home care

**Fig. 2:**
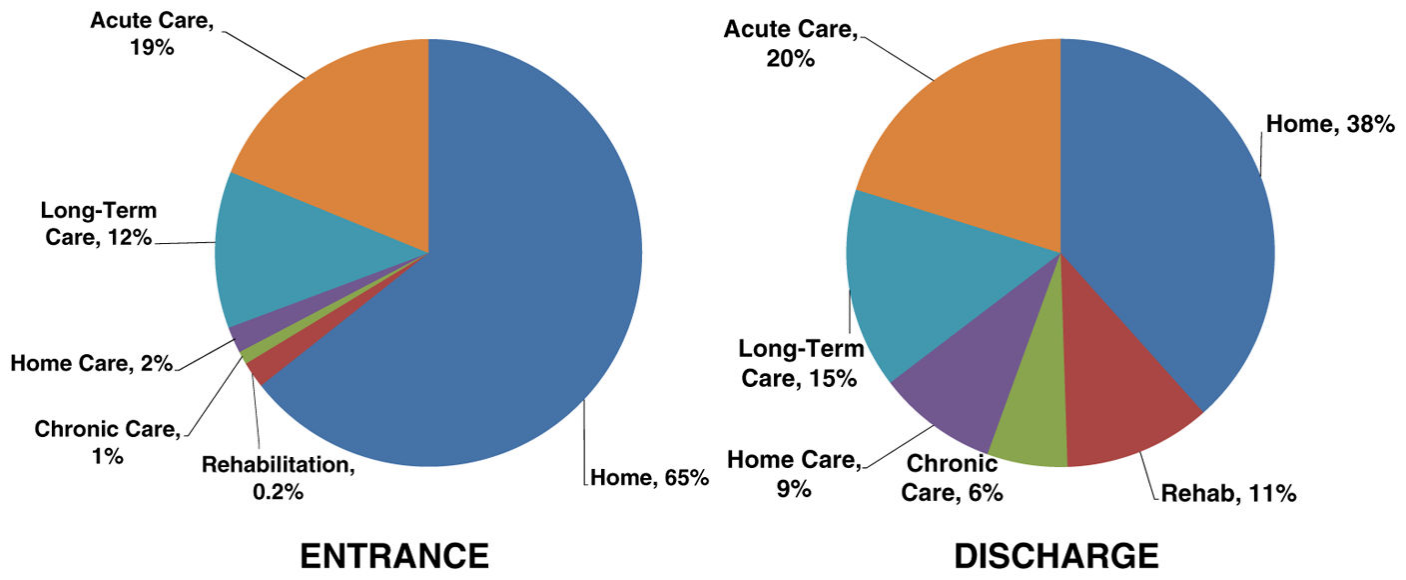
Entrance and discharge institutions following hospitalization for osteoporosis-related fracture (*N*=57,433)

Figure 2 presents the information used to calculate the net number of patients discharged to rehabilitation, continuing care, and chronic care (i.e., discharge location—entrance). For example, although 5,714 were discharged to rehabilitation facilities, 133 patients (78 hip fractures) were admitted from a rehabilitation facility to acute care for a net transfer of 5,581 individuals. While 15% of all hip fractures were discharged to rehabilitation facilities (*N*=4,284), hip fracture accounted for 75% of all discharges to rehabilitation facilities (*N*=4,284 out of 5,714). With an average cost per day of $736 and a total of 131,944 days spent in rehabilitation services, the cost associated with osteoporosis-related rehabilitation facilities was estimated to be over $97 million.

Similar calculations were used to determine the net number of individuals discharged to continuing care (*n*=2,391). Each individual spent on average 91 days in continuing care for a total of $113 million. Although 15% of hospitalized individuals were discharged to long-term care (*n*=8,707) for an average duration of 194 days, 12% of those (*n*=7,152) were already living in long-term care before being hospitalized. The cost associated with the net transfers to long-term care facilities was estimated at $28 million. Based on home care data from Ontario, we estimated that 50,398 Canadians received home care services following osteoporosis-related fractures at a cost of $245 million, of which 41% was due to hip fracture.

### Physician and prescription drug costs

According to IMS data, there were more than 2.3 million osteoporosis-related physician visits in 2008 for a total of $143 million. Visits to general practitioners accounted for 81% of all visits. Brogan estimates indicated that $391 million were spent in 2008 in osteoporosis-related medications. More than 70% of this cost was incurred by public plans ($278 million).

### Indirect costs

The number of days missed from work due to osteoporosis-related fractures was estimated at 3,123,298 days (12,013 full-time employment years) for individuals aged 50 to 69 years. Days spent in hospital or receiving home care services accounted for more than 90% of all days not available from work. When labor force participation rates were applied to this data, the costs associated with time loss from work was estimated at $46 million. Caregiver wage losses were calculated at $69 million, for a total of $115 million in indirect costs.

### Burden of osteoporosis: base case and sensitivity analyses

The base case estimates of the cost of osteoporosis in Canada in FY 2007/2008 were $2.3 billion (Table 4). Changing the rate of attribution to osteoporosis of fractures in women by using Quebec data rather than US data decreased the cost by 2%. Adding the cost associated with 2,096 cases with a most responsible diagnosis of osteoporosis alone increased the cost by 2%. In the sensitivity analysis assuming that 17% and 21% of men and women, respectively, were living in long-term care facilities due to osteoporosis, the total estimates increased to $3.9 billion (Table 4). In another sensitivity analysis assuming that all high and low-trauma fractures were due to osteoporosis, the base case estimates increased by 9% to $2.5 billion. Taken together, these results indicated that the upper bound of the burden of osteoporosis in Canada could be $4.1 billion when it was assumed that all trauma fractures were osteoporotic and that 17% of men and 21% of women over the age of 65 were admitted to long-term facilities due to osteoporosis.

## Discussion

In addition to the increased morbidity and mortality associated with fractures [25, 26], these results show that osteoporosis among Canadians aged 50 years and older is associated with a substantial economic cost accounting in 2008 for $2.3 billion or 1.3% of Canadian healthcare budget [27]. Specifically, our base case results indicated that osteoporosis was responsible for more than 57,413 hospitalizations and 832,594 hospitalized days in FY 2007/2008. This is more than the number of hospitalizations due to stroke (29,874 in FY 2007/2008) or heart attack (49,220 in FY 2007/2008) in Canada [28]. The acute care cost of managing these fractures was over $1.2 billion, or 50% of the total costs.

In contrast to the previous 1993 Canadian burden of illness study [4] which assumed that there were approximately 18,000 Canadians aged 75 years or over in long-term care facilities due to osteoporosis, our base case estimates did not include these individuals as the main reason of admission to long-term facilities could not be determined (e.g., hip fracture versus dementia with hip fracture as the sentinel event), making direct comparisons between the two studies difficult. However, when we included these individuals in a sensitivity analysis, the burden of illness estimate increased to $3.9 billion, which was approximately the double of the 1993 estimate expressed in 2010 dollars ($1.8 billion). Our cost estimates of the acute care treatment of osteoporosis-related fractures were also twice that of the 1993 estimates expressed in 2010 dollars ($1.2 billion versus $0.6 billion, respectively). Several reasons can explain these differences and caution should be exercised when comparing the 1993 and 2010 burden of illness estimates. First, the Canadian population aged 50 years and over has increased by 50% from 1993 to 2008, which may explain the increase in the number of hospitalized hip fractures between 1993 (*N*=21,302) and 2008 (*N*=28,867). Although the number of hospitalizations due to wrist fractures in Canada also increased from 2,149 to 4,858 during the same time period, the number of vertebral fractures decreased from 5,764 to 2,297. The use of a broader diagnostic code in the previous study to identify vertebral fractures may explain this difference. For example, the 1993 estimate of the number of vertebral fractures included fractures of the sacrum and coccyx, which were not considered in our study. Second, in addition to hip, wrist, and vertebral fractures, the costs associated with fractures of the humerus, multiple, and other sites were also included in our study while these fractures were not considered in determining the 1993 estimates. As such, it is more appropriate to compare the 1993 acute care costs (i.e., $0.6 billion in 2010 dollars) to the 2010 acute care costs associated with hip, wrist, and vertebral fractures only (i.e., $0.8 billion). Considering that the acute care costs associated with the other types of osteoporosis-related fractures accounted for 0.4 billion in our study, the 1993 acute care costs may have been an underestimation of the burden of osteoporosis. Interestingly enough, the 1993 average inpatient cost per hip fracture in 2010 dollars ($457 million for 21,233 hip fractures or an average of approximately $21,500 per hip fracture) was similar to our figure ($622 million for 28,267 hip fractures or approximately $21,600 per hip fracture). It was not possible to compare the average hospitalization/acute care cost per wrist or vertebral fracture between the two studies as the 1993 estimates included the outpatient costs associated with the management of wrist and vertebral fractures. Third, although the two studies were primarily based on CIHI data to estimate the acute care costs attributable to osteoporosis, different methods and data sources were used when estimating non-acute care costs. For example, we included the costs associated with rehabilitation and home care services which were not taken into consideration in the 1993 estimates. However, our long-term care cost estimates were double that of the 1993 study ($1.6 billion versus $0.8 billion, respectively) when we assumed that a proportion of individuals were living in long-term care due to osteoporosis (*N*=30,425 compared to *N*=19,900 in the 1993 study). This translated into an average of approximately $54,000 per long-term care resident in our study versus $38,000 in the previous study (in 2010 Canadian dollars). Another difference between the two studies relates to the higher costs of prescription drugs in our study (i.e., $391 million versus $20 million in 1993) which is consistent with the introduction of new treatment options for osteoporosis. Finally, our estimate of the physician costs attributable to osteoporosis was almost ten times higher than the 1993 estimates (i.e., $143 million versus $18 million in 1993). Difference in methods (e.g., expert opinion in the 1993 study versus IMS data in the 2010 study) may explain this difference.

Although it is difficult to directly compare our Canadian estimates with burden of illness studies conducted outside of Canada [29–37] due to differences in demographic variables (e.g., age, sex), methods (e.g., identification of osteoporosis-related fractures; cost categories included in estimates), or health care delivery systems (e.g., long-term care), our Canadian estimates were consistent with a recent US study which used a representative sample of Medicare to estimate the annual medical costs of osteoporosis in the elderly at $22 billion in 2008 [29]. Although the majority of burden of illness studies only reported the costs associated with osteoporosis-related hospitalizations [32, 34–36], non-acute care accounted for almost 50% of our base case direct cost estimates, which was higher than estimates reported in the US (38%) [37], Germany (33%) [30], and New Zealand (33%) [31]. Differences in the cost categories included in the non-acute care calculations may explain these variations (e.g., home care and long-term care). From a societal perspective, our results indicated that indirect costs accounted for 5% of the total costs, which was lower than an estimate from Germany (i.e., 15%) [30]. While we calculated indirect costs in terms of productivity losses and caregiver time loss due to treatment and rehabilitation of osteoporotic fractures, Brecht et al. [30] incorporated the unfitness for work, early retirement, and premature mortality in their calculations. As very few burden of illness studies have taken a societal perspective in their approach, determining the indirect costs associated with osteoporosis is an important area of future research.

Despite its strengths (e.g., patient-level data for many administrative datasets; national and provincial data), several limitations were associated with this study. First, the burden of osteoporosis in Quebec was estimated rather than derived from Quebec administrative data. However, our projected estimates of the number of osteoporosis-related fractures and associated inpatient costs in women were close to the estimates derived from the Recognizing Osteoporosis and its Consequences (ROCQ) Quebec cohort [22, 38]. Like other administrative data, there is always a risk of misclassification when reporting diagnostic information. For this reason, we excluded for the base case results those osteoporosis cases without a fracture or relevant intervention codes. Although we used the most responsible diagnosis at discharge to identify the population of study, some of the days spent in hospitals may be related to other conditions. In the absence of national data, we extrapolated provincial data to national levels by adjusting for differences in age and gender characteristics. However, we were not able to adjust for fracture types which may be different between provinces. However, little differences in hip fracture rates were observed between Canadian provinces [39]. We also used provincial unit costs assuming that the data may be representative of other Canadian provinces, which may not be true. However, we found very little variation in the average value of the RIWs between Canadian provinces (less than 5%). Similarly in the absence of data, the costs associated with primary and community care of fractures were not captured in our analyses (e.g., vertebral fractures most commonly treated in outpatient settings), which may result in an underestimation of the true cost of osteoporosis in Canada. In addition, the costs of therapy may have been underestimated as calcium and vitamin D supplementation costs were not included in our estimates or the costs associated with premature mortality. In the absence of data, we also determined the rate of attribution to osteoporosis for non-hip non-vertebral fractures to match Mackey’s estimates, which may have introduced some bias in our calculations. However, the results changed little when Quebec data were used for the attribution rate of osteoporosis in women [22]. Finally we excluded fractures at sites that are not typically related to osteoporosis, such as fractures of the heel, toe, hand, finger, face, or skull.

In conclusion, the burden of osteoporosis in FY 2007/2008 was estimated to range from $2.3 billion to $4.1 billion. Since the prevalence of osteoporosis increases with age, the burden of osteoporosis is likely to increase over the next decade. As such, prevention of osteoporotic fractures among patients at high risk of fractures is key to decreasing the human and economic burden of osteoporosis. Future research should continue to provide detailed information on the burden of osteoporosis by gender, age group, and fracture type that could be used for resource allocation and prioritization.

## Figures and Tables

**Table 1:** Unit costs, data sources, and main costing assumptions

Cost component	Item	Unit costs (data source)	Main costing assumptions
Acute care (includes acute care bed admissions, emergency room visits, day surgeries— with identical methodology)	Cost per RIW	$5,399.04 (CIHI)	Quebec hospitalizations extrapolated from all other Canadian provinces Ontario data on number of same day surgeries and emergency room visits extrapolated to Canada Patient-level costing
	Physician visit fees^a^	$79.20 (admission); $55.45 (2nd, 3rd, and last day); $29.20 (other days) (OSBPS)	
	Diagnostic tests	Range from $33 for wrist X-ray to $117 for MRI of vertebral fracture (average of $75) (OSBPS)	
	Surgeon, surgical assistant, and anesthesiologist procedure fees for assessment, procedure, and follow-up	Range from $76 immobilization of hip to $2,551 for fixation or reduction for vertebral fracture (average $1,352) (OSBPS)	
Rehabilitation	Cost per RIW per stay	$15,449 (CIHI)	Based on net transfers from acute care Length of stay and costing based on rehabilitation database Patient-level costing
Continuing care	Cost per RIW per day	$420.12 (CIHI)	Based on net transfers from acute care Length of stay and costing based on continuing database Patient-level costing
Home care	Cost per week	$168.50 (MDS Inter-rai)	Ontario data on number of recipients extrapolated to Canada Length of stay based on Manitoba data and unit costs from Ontario
Long-term care	Cost per day	$147.77 (Ontario provincial budget)	Based on net transfers from acute care Length of stay based on Manitoba data and unit costs from Ontario
Outpatient physician services	Physician visit fees	General practice: consultation (1 per year) $56.10, repeat consultation $42.35 Internal medicine: consultation $132.50, repeat consultation $82.90	Assume 50% of visits are consultation and 50% are repeat consultations
Drug costs	National estimates from public and private plans	Retail drug price as charged, plus $7.00 dispensing fee (IMS Brogan PharmaStat©)	100% of public data programs covered in most provinces (except PEI and Social Services in Alberta) Over 65% of all national privately reimbursed prescriptions
Productivity losses	Cost per day	$24.12 per hour×8 h per day (Statistics Canada)	Number of days based on CAMOS data

*RIW* resource intensity weight, *CIHI* Canadian Institute for Health Information, *OSBPS* Ontario Schedule of Benefits for Physician Services, *MDS Inter-rai* minimal data set

a For example, fees associated with orthopedic surgeons, anesthesiologists, and radiologists as not included in RIW

IMS Brogan data request: http://www.store.imshealth.com/

**Table 2:** Canadian hospitalizations and hospitalized days for osteoporosis-related fractures by gender and type of fracture (fiscal year 2007/2008)

Fracture type	Number of hospitalizations: all Canada except Quebec			Number of hospitalizations: Quebec (extrapolated)			Number of hospitalizations: Canada	Number of hospitalized days: Canada
	Women	Men	Total	Women	Men	Total	Total	Total
Hip	15,519	6,165	21,684	5,156	2,027	7,183	28,867	448,776
Humerus	1,623	4,54	2,077	397	111	508	2,585	30,141
Vertebral	1,077	6,45	1,722	360	215	575	2,297	34,490
Wrist	2,983	9,37	3,920	714	224	938	4,858	24,132
Multiple site	3,325	1,579	4,904	762	362	1,124	6,028	124,000
Other site	7,034	3,366	10,400	1,608	770	2,378	12,778	171,055
Total	31,561	13,146	44,707	8,997	3,709	12,709	57,413	832,594

**Table 3:** Acute care costs (2010 Canadian dollars)

Fracture type	Emergency care	Acute care admissions	Same day surgery	Total acute care costs	Percent of total costs
Hip	$40,493,177	$582,058,662	$288,169	$622,840,008	53%
Humerus	$11,681,974	$32,324,504	$451,514	$44,457,992	4%
Vertebral	$5,186,182	$31,720,622	$237,393	$37,144,197	3%
Wrist	$55,420,934	$43,028,096	$9,497,406	$107,946,436	9%
Multiple sites	$9,322,424	$141,035,749	$321,292	$150,679,465	13%
Other sites	$38,803,610	$178,163,216	$1,239,783	$218,206,609	18%
Total	$160,908,302	$1,008,330,849	$12,035,556	$1,181,274,707	100%
Percent of total costs	14%	85%	1%	100%	
Percent of total costs attributed to women	72%	71%	76%	71%	

**Table 4:** Burden of osteoporosis: base case and sensitivity analyses (2010 Canadian dollars)

Cost component	Base case analysis	Change attribution rates of osteoporosis using ROCQ data instead of MacKey et al.	Add costs attributed to hospitalizations due to osteoporosis only (*N*=2,096)	Assumes that a proportion of long-term care residents were admitted due to osteoporosis-related fractures	Assumes that all high and low-trauma fractures are osteoporotic
Acute care costs (hospitalization, same day surgeries, and emergency room visits)	$1,181,274,707	$1,134,803,061	$1,219,450,008	Unchanged	$1,318,689,391
Rehabilitation costs	$97,169,606	$95,280,270	$103,457,541	Unchanged	$120,170,851
Continuing care costs	$112,720,625	$110,024,143	$119,837,738	Unchanged	$140,969,693
Long-term care	$28,275,046	$26,487,393	Unchanged	$1,641,017,974	$46,532,134
Home care services	$244,565,735	Unchanged	Unchanged	Unchanged	Unchanged
Physician costs	$142,589,880	Unchanged	Unchanged	Unchanged	Unchanged
Prescribed drug costs	$390,854,843	Unchanged	Unchanged	Unchanged	Unchanged
Indirect costs	$115,311,966	$115,045,033	Unchanged	Unchanged	$117,076,070
Total cost	$2,312,762,408	$2,263,759,530	$2,364,342,757	$3,925,505,337	$2,519,684,494

*ROCQ* Recognizing Osteoporosis and its Consequences

## Appendix 1

**Table 5:** List of ICD-10 CA codes by type of fracture

Fracture type	ICD 10 codes relating to fracture type
Hip	S72.0, S72.1, S72.2
Humerus	S42.2
Vertebral	S22.0, S22.1, S32.0
Wrist	S52 with CCI codes
Other sites:	
• Femur	S72.3, S72.4, S72.7, S72.8, S72.9
• Lower leg (tibia, fibula, ankle, knee, foot)	S82.0–S82.9, S92
• Lower arm (radius, ulna)	S52 *unless wrist above*
• Other site (rib, shoulder, arm)	S22.3, S42.0, S42.7, S42.8, S42.9
• Other fractures including:	S22.2, S22.4, S22.8, S22.9
• ribs/sternum, clavicle, pelvis, patella,	S32.1, S32.3, S32.4, S32.5, S32.7, S32.8
• tibia/fibula, ankle	S42.0–42.9 except 42.2, S42.7, S42.8, S42.9 S72.0–72.9 *except when* “*hip/femur*” *from above*
Multiple fractures	T02.1–T02.9 (*or more than 1 of above*)
